# A Case of an Apical Aneurysm Caused by a Cardiac Event Involving Noncoronary Collateral Blood Flow

**DOI:** 10.7759/cureus.43520

**Published:** 2023-08-15

**Authors:** Ryoetsu Yamanaka

**Affiliations:** 1 Cardiology, Kyoto Yamashiro General Medical Center, Kyoto, JPN

**Keywords:** heart failure, minoca, nccmbf, inferior phrenic artery, ventricular aneurysm

## Abstract

An 87-year-old man presented with exertional dyspnea and was admitted due to congestive heart failure. Echocardiography and left ventriculography performed after his condition improved showed an aneurysm at the left ventricular apex. However, coronary angiography showed no significant lesions and an avascular field at the apex. Computed tomography angiography revealed that the enlarged left inferior phrenic artery reached the heart and nourished the apex wall where the aneurysm was present. Looking back retrospectively, he was previously hospitalized nine years ago for epigastric pain with elevated myocardial deviation enzymes and electrocardiographic changes but no coronary artery lesions. Moreover, abnormal vascularization had already been observed 13 years ago when the aneurysm did not exit. Considering these findings, we concluded that the ventricular aneurysm in this case was caused by a vascular event involving collateral circulation from outside the heart.

## Introduction

Albeit infrequent, reports have recognized the presence of collateral flow that nourishes the heart from outside, a phenomenon called "noncoronary collateral myocardial blood flow" (NCCMBF), with several previous reports on this phenomenon having been published [1-4]. However, there have been no case reports of ischemic events such as acute coronary syndrome involving NCCMBF. On the other hand, ischemic heart disease in which the culprit artery cannot be identified certainly exists, so it may be that such cases have not been detected. We encountered a case in which NCCMBF was presumed to have caused a cardiovascular event, resulting in a ventricular aneurysm. Herein, we detail our experience along with the relevant literature.

## Case presentation

**Figure 1:**
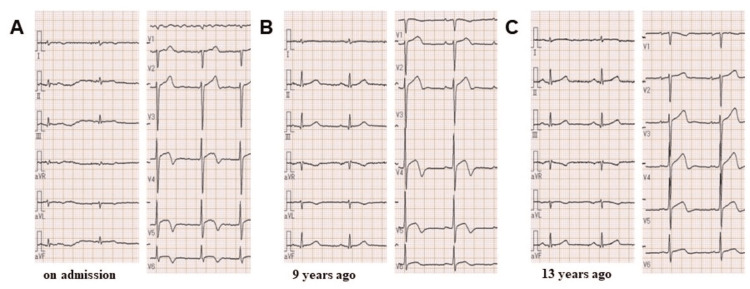
Electrocardiogram transition. (A) On admission: biphasic T-waves were observed in the chest leads; (B) 9 years ago: similarly, biphasic T-waves were observed; (C) 13 years ago: no abnormality was yet observed.

**Figure 2:**
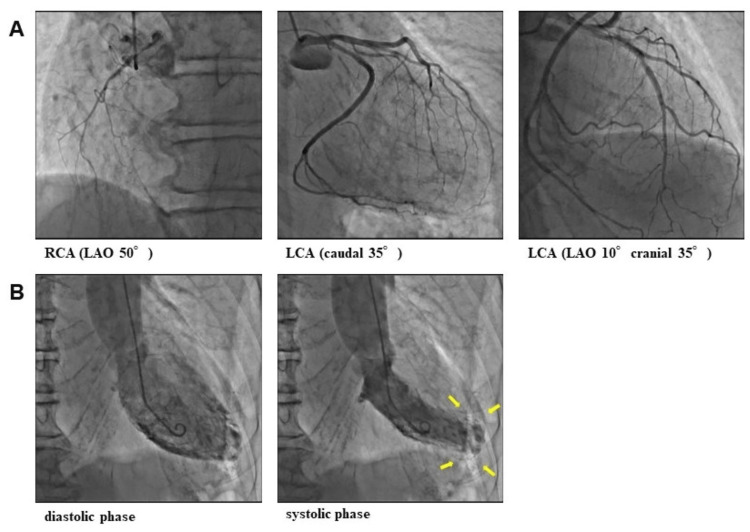
Coronary angiography and left ventriculography. (A) No lesions were detected in the coronary arteries; (B) a small aneurysm was formed in the apex wall on left ventriculography (arrows).

**Figure 3:**
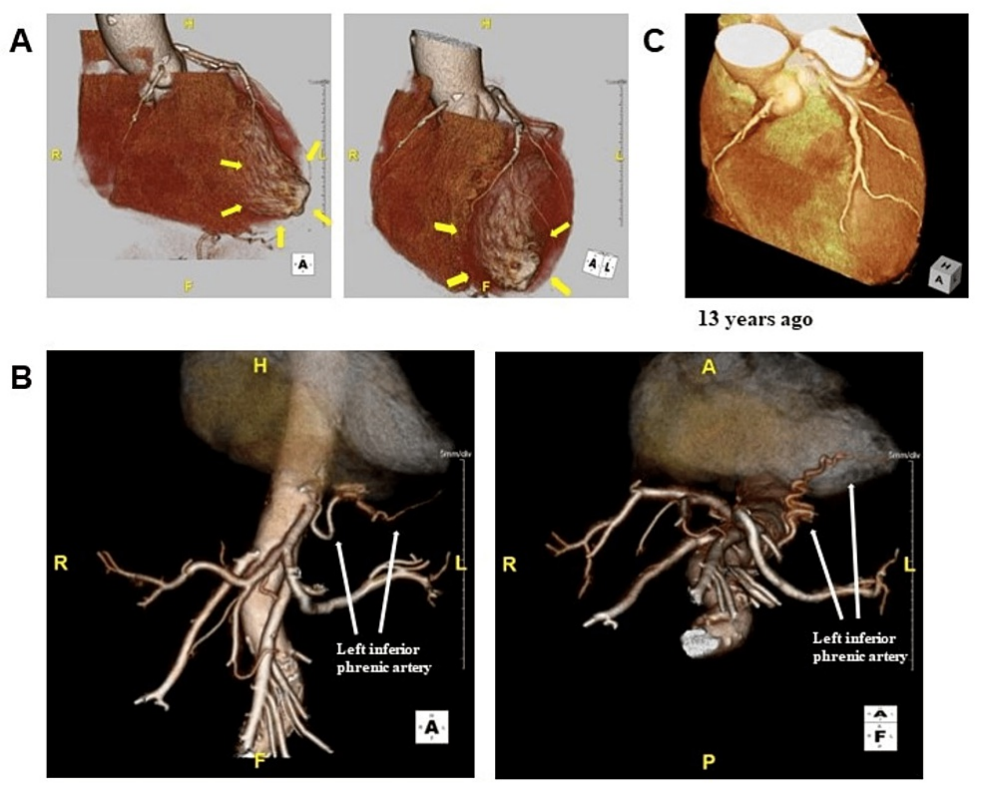
Computed tomography angiography. (A) No lesions in the coronary arteries consistent with coronary angiography. However, the apex with aneurysm appeared to be looked like an avascular field (arrows). (B) The left inferior phrenic artery, which branches directly from the descending aorta, was markedly dilated to nourish the apex of heart. (C) 13 years ago: the arrangement of vessels had not changed before the formation of the aneurysm.

An 87-year-old man was referred to the emergency department of our hospital with a complaint of dyspnea on exertion (New York Heart Association functional class Ⅲ) starting a week ago. This patient’s medical history included internal carotid artery stenosis (untreated and under follow-up), dyslipidemia, diabetes, and paroxysmal atrial fibrillation. Moreover, the patient had been hospitalized for epigastric discomfort nine years ago and discharged after several days because his symptoms improved without any treatment, although the cause was unknown. His medications included rivaroxaban (10 mg/day), clopidogrel (75 mg/day), bisoprolol (2.5 mg/day), and rosuvastatin (2.5 mg/day). At admission, he had a blood pressure of 131/76 mmHg, a pulse rate of 58/min, and an oxygen saturation of 92% (without oxygen administration). His physical examination revealed an irregular heart rhythm with no significant murmur, normal respiratory sounds in both lungs, and pitting edema in both lower extremities. Initial laboratory tests showed a hemoglobin level of 13.8 g/dL (standard range: 13.5-17.6 g/dL), a creatinine level of 1.01 mg/dL (standard range: 0.40-1.10 mg/dL), an estimated glomerular filtration rate of 52 mL/min/1.73 m^2^, a brain natriuretic peptide level of 338.5 pg/mL (standard range: 0.0-18.4 pg/mL), and a troponin I level of 0 ng/mL (standard range: 0-0.04 ng/mL). His 12-lead electrocardiography revealed atrial fibrillation and a biphasic T wave in the V4-V6 lead (Figure 1A), whereas his chest X-ray showed enlargement of the cardiothoracic ratio and dullness of both costophrenic angles. On initial transthoracic echocardiography, akinesis with wall thinning was observed located in the anterior-apical wall of the left ventricle. Other left ventricular wall contractions were normal, and the left ventricular end-systolic/end-diastolic volume and ejection fraction were 69.6 ml, 30.0 ml, and 56.9%, respectively. In addition, it revealed mild left atrial enlargement (left atrial volume index = 36.8 ml/m^2^) and mild-moderate regurgitations of the mitral and tricuspid valves. The inferior vena cava was dilated, and respiratory fluctuations disappeared. Based on the mentioned results, the patient was diagnosed with an acute exacerbation of heart failure and hospitalized for treatment. A single intravenous injection of furosemide (40 mg/day) rapidly improved his symptoms and objectionable findings. After compensation for heart failure, cardiac catheterization was performed to determine the presence of underlying heart disease. Accordingly, coronary angiography (CAG) revealed that the left anterior descending artery (LAD) was unusually shorter without occlusion and that the anterior apex wall was an avascular area. No significant stenosis was identified (Figure 2A). Left ventriculography found a left ventricular aneurysm at the anterior apex wall similar to that found during echocardiography (Figure 2B). To investigate the cause of the apical aneurysm, cardiac computed tomography (CT) angiography was performed after a few days. The coronary arteries were the same as during CAG, whereas the left inferior phrenic artery was significantly developed and supplied the anterior apex of the left ventricle, which seemed to be an avascular field, through the diaphragm (Figure 3A-3B). Retrospectively, the pathway of this artery had already been confirmed on contrast-enhanced CT, which was conducted for another purpose 13 years ago (Figure 3C). At that time, however, the apex aneurysm had not been detected. T-wave abnormalities in the V4-V6 lead had occurred since admission for unexplained epigastric pain nine years ago (Figure 1B-1C), during which a slight increase in myocardial deviant enzymes, such as creatine kinase, had also been observed. Therefore, the apical aneurysm was speculated to have been present nine years ago due to a cardiac event involving the left inferior phrenic artery as the culprit lesion.

## Discussion

We described a case of an apical aneurysm formed through a vascular event involving collateral circulation from outside the heart.

In general, reports have shown that the majority of apical aneurysms result from obstruction of the distal LAD [5]. In the current case, we initially suspected an old myocardial infarction in the LAD area. However, doubts emerged after having determined that the aneurysm was so small and localized to the anterior wall side, which did not match the perfusion area of the LAD. Moreover, the coronary arteries, including the LAD, remain unobstructed but did not reach the anterior apex wall, which was the site of the aneurysm, whereas the epicardium of the anterior apex is nourished by a growing left inferior phrenic artery. This vascular arrangement has remained the same since before the aneurysm was formed, suggesting that the blood flow from the phrenic artery was not caused by the obstruction of other vessels, such as the LAD. Given that the patient developed epigastric pain with electrocardiogram changes and elevated myocardial deviation enzymes nine years ago, we assumed that this episode had been a cardiovascular event that resulted in the apical aneurysm. At that time, the cause of the symptom remained unknown, and the patient was discharged from the hospital early. Thus, investigating the detailed mechanism is no longer possible. Nonetheless, our findings suggest that the culprit lesion for the event was most likely the inferior phrenic artery.

Similar to the phrenic artery in this case, blood flow that reaches the myocardium from outside the heart has been called NCCMBF or "noncoronary collateral circulation" (NCCC), with several previous reports on the phenomenon having been published [1-4]. Moreover, studies have shown that NCCMBF can arise from various arteries, such as the bronchial, esophageal, pericardial, diaphragmatic, and aortic arteries [3], and its vessel diameter can be less than 1 mm or more than 3 mm [6]. Furthermore, NCCMBF is said to be present in 20% of all humans, especially in 50% of patients with ischemic heart disease [6]. Although NCCMBF is estimated to have approximately one-fifth of the blood flow of the coronary arteries [7], few reports have demonstrated related cardiovascular events.

Myocardial infarction with non-obstructive coronary arteries (MINOCA) has continued to attract attention, with its detailed mechanism yet to be elucidated [8,9]. Some cases of MINOCA may involve cardiovascular events in the NCCMBF, such as in the current case.

## Conclusions

We report a rare case of an apical aneurysm caused by NCCMBF originating from the inferior phrenic artery. Furthermore, no data are currently available regarding an aneurysm formed through a vascular event involving collateral circulation from outside the heart. The presence of extracardiac collateral circulation may be considered in cases where the culprit lesion of acute coronary syndrome cannot be identified. Furthermore, cardiac events from extracardiac blood flow may be considered one of the causes of MINOCA.
